# Supplementary material for: Joint profiling of cell morphology and gene expression during in vitro neurodevelopment
Source: eLife. 2025 Dec 1;14:e102578. doi: 10.7554/eLife.102578 (PMC12668676; doi:10.7554/eLife.102578)
Supplement: Supplementary file 2. — Formula of transformation applied per CP feature, ordered by feature families. [file elife-102578-supp2.pdf]

feature name	transfo'	feature name	transfo'	feature name	transfo'	feature name	transformation
Cel_Shape_Eccentricity	x	Nuc_Txtr_Entropy_Nuc_3_00_256	x	Cyt_Int_IntegrIntEdge_CGAN	log2(x+1)	Nuc_Shape_MinFeretDiameter	log2(x+1)
Cel_Shape_Extent	x	Nuc_Txtr_Entropy_Nuc_3_01_256	x	Cyt_Int_IntegrIntEdge_EndoRet	log2(x+1)	Nuc_Shape_MinorAxisLength	log2(x+1)
Cel_Shape_Solidity	x	Nuc_Txtr_Entropy_Nuc_3_02_256	x	Cyt_Int_IntegrIntEdge_Mito	log2(x+1)	Cyt_Txtr_SumAvg_CGAN_3_00_256	log2(x+1)
Cel_Shape_Zernike_0_0	x	Nuc_Txtr_Entropy_Nuc_3_03_256	x	Cyt_Int_IntegratedInt_CGAN	log2(x+1)	Cyt_Txtr_SumAvg_CGAN_3_01_256	log2(x+1)
Cel_Shape_Zernike_1_1	x	Nuc_Txtr_Corr_Nuc_3_00_256	x	Cyt_Int_IntegratedInt_EndoRet	log2(x+1)	Cyt_Txtr_SumAvg_CGAN_3_02_256	log2(x+1)
Cel_Shape_Zernike_2_0	x	Nuc_Txtr_Corr_Nuc_3_01_256	x	Cyt_Int_IntegratedInt_Mito	log2(x+1)	Cyt_Txtr_SumAvg_CGAN_3_03_256	log2(x+1)
Cel_Shape_Zernike_2_2	x	Nuc_Txtr_Corr_Nuc_3_02_256	x	Cyt_Int_LowerQInt_CGAN	log2(x+1)	Cyt_Txtr_SumAvg_EndoRet_3_00_256	log2(x+1)
Cel_Shape_Zernike_3_1	x	Nuc_Txtr_Corr_Nuc_3_03_256	x	Cyt_Int_LowerQInt_EndoRet	log2(x+1)	Cyt_Txtr_SumAvg_EndoRet_3_01_256	log2(x+1)
Cel_Shape_Zernike_3_3	x	Cyt_Txtr_InvDiffMnt_CGAN_3_00_256	x	Cyt_Int_LowerQInt_Mito	log2(x+1)	Cyt_Txtr_SumAvg_EndoRet_3_02_256	log2(x+1)
Cel_Shape_Zernike_4_0	x	Cyt_Txtr_InvDiffMnt_CGAN_3_01_256	x	Cyt_Int_MADInt_CGAN	log2(x+1)	Cyt_Txtr_SumAvg_EndoRet_3_03_256	log2(x+1)
Cel_Shape_Zernike_4_2	x	Cyt_Txtr_InvDiffMnt_CGAN_3_02_256	x	Cyt_Int_MADInt_EndoRet	log2(x+1)	Cyt_Txtr_SumAvg_Mito_3_00_256	log2(x+1)
Cel_Shape_Zernike_4_4	x	Cyt_Txtr_InvDiffMnt_CGAN_3_03_256	x	Cyt_Int_MADInt_Mito	log2(x+1)	Cyt_Txtr_SumAvg_Mito_3_01_256	log2(x+1)
Cel_Shape_Zernike_5_1	x	Cyt_Txtr_InvDiffMnt_EndoRet_3_00_256	x	Cyt_Int_MassDisplacement_CGAN	log2(x+1)	Cyt_Txtr_SumAvg_Mito_3_02_256	log2(x+1)
Cel_Shape_Zernike_5_3	x	Cyt_Txtr_InvDiffMnt_EndoRet_3_01_256	x	Cyt_Int_MassDisplacement_EndoRet	log2(x+1)	Cyt_Txtr_SumAvg_Mito_3_03_256	log2(x+1)
Cel_Shape_Zernike_5_5	x	Cyt_Txtr_InvDiffMnt_EndoRet_3_02_256	x	Cyt_Int_MassDisplacement_Mito	log2(x+1)	Nuc_Txtr_SumAvg_Nuc_3_00_256	log2(x+1)
Cel_Shape_Zernike_6_0	x	Cyt_Txtr_InvDiffMnt_EndoRet_3_03_256	x	Cyt_Int_MaxIntEdge_CGAN	log2(x+1)	Nuc_Txtr_SumAvg_Nuc_3_01_256	log2(x+1)
Cel_Shape_Zernike_6_2	x	Cyt_Txtr_InvDiffMnt_Mito_3_00_256	x	Cyt_Int_MaxIntEdge_EndoRet	log2(x+1)	Nuc_Txtr_SumAvg_Nuc_3_02_256	log2(x+1)
Cel_Shape_Zernike_6_4	x	Cyt_Txtr_InvDiffMnt_Mito_3_01_256	x	Cyt_Int_MaxIntEdge_Mito	log2(x+1)	Nuc_Txtr_SumAvg_Nuc_3_03_256	log2(x+1)
Cel_Shape_Zernike_6_6	x	Cyt_Txtr_InvDiffMnt_Mito_3_02_256	x	Cyt_Int_MaxInt_CGAN	log2(x+1)	Cel_Neighbors_ScndClosestDist_Adjacent	log2(x+1)
Cel_Shape_Zernike_7_1	x	Cyt_Txtr_InvDiffMnt_Mito_3_03_256	x	Cyt_Int_MaxInt_EndoRet	log2(x+1)	Cel_Neighbors_FrstClosestDist_Adjacent	log2(x+1)
Cel_Shape_Zernike_7_3	x	Nuc_Txtr_InvDiffMnt_Nuc_3_00_256	x	Cyt_Int_MaxInt_Mito	log2(x+1)	Cel_Shape_EulerNumber	log2(max(x)-x+0.1)
Cel_Shape_Zernike_7_5	x	Nuc_Txtr_InvDiffMnt_Nuc_3_01_256	x	Cyt_Int_MeanIntEdge_CGAN	log2(x+1)	Cel_Neighbors_AngleBtNghbors_Adjacent	2{ log2(max(x)-x+1)}
Cel_Shape_Zernike_7_7	x	Nuc_Txtr_InvDiffMnt_Nuc_3_02_256	x	Cyt_Int_MeanIntEdge_EndoRet	log2(x+1)	Cyt_Txtr_Contrast_CGAN_3_00_256	log2(x+0.01)
Cel_Shape_Zernike_8_0	x	Nuc_Txtr_InvDiffMnt_Nuc_3_03_256	x	Cyt_Int_MeanIntEdge_Mito	log2(x+1)	Cyt_Txtr_Contrast_CGAN_3_01_256	log2(x+0.01)
Cel_Shape_Zernike_8_2	x	Nuc_Shape_Eccentricity	x	Cyt_Int_MeanInt_CGAN	log2(x+1)	Cyt_Txtr_Contrast_CGAN_3_02_256	log2(x+0.01)
Cel_Shape_Zernike_8_4	x	Nuc_Shape_Extent	x	Cyt_Int_MeanInt_EndoRet	log2(x+1)	Cyt_Txtr_Contrast_CGAN_3_03_256	log2(x+0.01)
Cel_Shape_Zernike_8_6	x	Nuc_Shape_Solidity	x	Cyt_Int_MeanInt_Mito	log2(x+1)	Cyt_Txtr_Contrast_EndoRet_3_00_256	log2(x+0.01)
Cel_Shape_Zernike_8_8	x	Cyt_Txtr_SumEntropy_CGAN_3_00_256	x	Cyt_Int_MedInt_CGAN	log2(x+1)	Cyt_Txtr_Contrast_EndoRet_3_01_256	log2(x+0.01)
Cel_Shape_Zernike_9_1	x	Cyt_Txtr_SumEntropy_CGAN_3_01_256	x	Cyt_Int_MedInt_EndoRet	log2(x+1)	Cyt_Txtr_Contrast_EndoRet_3_02_256	log2(x+0.01)
Cel_Shape_Zernike_9_3	x	Cyt_Txtr_SumEntropy_CGAN_3_02_256	x	Cyt_Int_MedInt_Mito	log2(x+1)	Cyt_Txtr_Contrast_EndoRet_3_03_256	log2(x+0.01)
Cel_Shape_Zernike_9_5	x	Cyt_Txtr_SumEntropy_CGAN_3_03_256	x	Cyt_Int_MinIntEdge_CGAN	log2(x+1)	Cyt_Txtr_Contrast_Mito_3_00_256	log2(x+0.01)
Cel_Shape_Zernike_9_7	x	Cyt_Txtr_SumEntropy_EndoRet_3_00_256	x	Cyt_Int_MinIntEdge_EndoRet	log2(x+1)	Cyt_Txtr_Contrast_Mito_3_01_256	log2(x+0.01)
Cel_Shape_Zernike_9_9	x	Cyt_Txtr_SumEntropy_EndoRet_3_01_256	x	Cyt_Int_MinIntEdge_Mito	log2(x+1)	Cyt_Txtr_Contrast_Mito_3_02_256	log2(x+0.01)
Nuc_Shape_Zernike_0_0	x	Cyt_Txtr_SumEntropy_EndoRet_3_02_256	x	Cyt_Int_MinInt_CGAN	log2(x+1)	Cyt_Txtr_Contrast_Mito_3_03_256	log2(x+0.01)
Nuc_Shape_Zernike_1_1	x	Cyt_Txtr_SumEntropy_EndoRet_3_03_256	x	Cyt_Int_MinInt_EndoRet	log2(x+1)	Nuc_Txtr_Contrast_Nuc_3_00_256	log2(x+0.01)
Nuc_Shape_Zernike_2_0	x	Cyt_Txtr_SumEntropy_Mito_3_00_256	x	Cyt_Int_MinInt_Mito	log2(x+1)	Nuc_Txtr_Contrast_Nuc_3_01_256	log2(x+0.01)
Nuc_Shape_Zernike_2_2	x	Cyt_Txtr_SumEntropy_Mito_3_01_256	x	Cyt_Int_StdIntEdge_CGAN	log2(x+1)	Nuc_Txtr_Contrast_Nuc_3_02_256	log2(x+0.01)
Nuc_Shape_Zernike_3_1	x	Cyt_Txtr_SumEntropy_Mito_3_02_256	x	Cyt_Int_StdIntEdge_EndoRet	log2(x+1)	Nuc_Txtr_Contrast_Nuc_3_03_256	log2(x+0.01)
Nuc_Shape_Zernike_3_3	x	Cyt_Txtr_SumEntropy_Mito_3_03_256	x	Cyt_Int_StdIntEdge_Mito	log2(x+1)	Cyt_Txtr_InfoMeas1_CGAN_3_00_256	log2(max(x)-x+0.5)
Nuc_Shape_Zernike_4_0	x	Nuc_Txtr_SumEntropy_Nuc_3_00_256	x	Cyt_Int_StdInt_CGAN	log2(x+1)	Cyt_Txtr_InfoMeas1_CGAN_3_01_256	log2(max(x)-x+0.5)
Nuc_Shape_Zernike_4_2	x	Nuc_Txtr_SumEntropy_Nuc_3_01_256	x	Cyt_Int_StdInt_EndoRet	log2(x+1)	Cyt_Txtr_InfoMeas1_CGAN_3_02_256	log2(max(x)-x+0.5)
Nuc_Shape_Zernike_4_4	x	Nuc_Txtr_SumEntropy_Nuc_3_02_256	x	Cyt_Int_StdInt_Mito	log2(x+1)	Cyt_Txtr_InfoMeas1_CGAN_3_03_256	log2(max(x)-x+0.5)
Nuc_Shape_Zernike_5_1	x	Nuc_Txtr_SumEntropy_Nuc_3_03_256	x	Cyt_Int_UpperQInt_CGAN	log2(x+1)	Cyt_Txtr_InfoMeas1_EndoRet_3_00_256	log2(max(x)-x+0.5)
Nuc_Shape_Zernike_5_3	x	Cel_Shape_Area	log2(x+1)	Cyt_Int_UpperQInt_EndoRet	log2(x+1)	Cyt_Txtr_InfoMeas1_EndoRet_3_01_256	log2(max(x)-x+0.5)
Nuc_Shape_Zernike_5_5	x	Cel_Shape_BBoxArea	log2(x+1)	Cyt_Int_UpperQInt_Mito	log2(x+1)	Cyt_Txtr_InfoMeas1_EndoRet_3_02_256	log2(max(x)-x+0.5)
Nuc_Shape_Zernike_6_0	x	Cel_Shape_Compactness	log2(x+1)	Nuc_Int_IntegrIntEdge_Nuc	log2(x+1)	Cyt_Txtr_InfoMeas1_EndoRet_3_03_256	log2(max(x)-x+0.5)
Nuc_Shape_Zernike_6_2	x	Cel_Shape_ConvexArea	log2(x+1)	Nuc_Int_IntegratedInt_Nuc	log2(x+1)	Cyt_Txtr_InfoMeas1_Mito_3_00_256	log2(max(x)-x+0.5)
Nuc_Shape_Zernike_6_4	x	Cel_Shape_EquivDiameter	log2(x+1)	Nuc_Int_LowerQInt_Nuc	log2(x+1)	Cyt_Txtr_InfoMeas1_Mito_3_01_256	log2(max(x)-x+0.5)
Nuc_Shape_Zernike_6_6	x	Cel_Shape_FormFactor	log2(x+1)	Nuc_Int_MADInt_Nuc	log2(x+1)	Cyt_Txtr_InfoMeas1_Mito_3_02_256	log2(max(x)-x+0.5)
Nuc_Shape_Zernike_7_1	x	Cel_Shape_MajorAxisLength	log2(x+1)	Nuc_Int_MassDisplacement_Nuc	log2(x+1)	Cyt_Txtr_InfoMeas1_Mito_3_03_256	log2(max(x)-x+0.5)
Nuc_Shape_Zernike_7_3	x	Cel_Shape_MaxFeretDiameter	log2(x+1)	Nuc_Int_MaxIntEdge_Nuc	log2(x+1)	Cyt_Txtr_InfoMeas2_CGAN_3_00_256	log2(max(x)-x+0.01)
Nuc_Shape_Zernike_7_5	x	Cel_Shape_MaxRadius	log2(x+1)	Nuc_Int_MaxInt_Nuc	log2(x+1)	Cyt_Txtr_InfoMeas2_CGAN_3_01_256	log2(max(x)-x+0.01)
Nuc_Shape_Zernike_7_7	x	Cel_Shape_MeanRadius	log2(x+1)	Nuc_Int_MeanIntEdge_Nuc	log2(x+1)	Cyt_Txtr_InfoMeas2_CGAN_3_02_256	log2(max(x)-x+0.01)
Nuc_Shape_Zernike_8_0	x	Cel_Shape_MedRadius	log2(x+1)	Nuc_Int_MeanInt_Nuc	log2(x+1)	Cyt_Txtr_InfoMeas2_CGAN_3_03_256	log2(max(x)-x+0.01)
Nuc_Shape_Zernike_8_2	x	Cel_Shape_MinFeretDiameter	log2(x+1)	Nuc_Int_MedInt_Nuc	log2(x+1)	Cyt_Txtr_InfoMeas2_EndoRet_3_00_256	log2(max(x)-x+0.01)
Nuc_Shape_Zernike_8_4	x	Cel_Shape_MinorAxisLength	log2(x+1)	Nuc_Int_MinIntEdge_Nuc	log2(x+1)	Cyt_Txtr_InfoMeas2_EndoRet_3_01_256	log2(max(x)-x+0.01)
Nuc_Shape_Zernike_8_6	x	Cel_Shape_Perimeter	log2(x+1)	Nuc_Int_MinInt_Nuc	log2(x+1)	Cyt_Txtr_InfoMeas2_EndoRet_3_02_256	log2(max(x)-x+0.01)
Nuc_Shape_Zernike_8_8	x	Cel_Int_IntegrIntEdge_CGAN	log2(x+1)	Nuc_Int_StdIntEdge_Nuc	log2(x+1)	Cyt_Txtr_InfoMeas2_EndoRet_3_03_256	log2(max(x)-x+0.01)
Nuc_Shape_Zernike_9_1	x	Cel_Int_IntegrIntEdge_EndoRet	log2(x+1)	Nuc_Int_StdInt_Nuc	log2(x+1)	Cyt_Txtr_InfoMeas2_Mito_3_00_256	log2(max(x)-x+0.01)
Nuc_Shape_Zernike_9_3	x	Cel_Int_IntegrIntEdge_Mito	log2(x+1)	Nuc_Int_UpperQInt_Nuc	log2(x+1)	Cyt_Txtr_InfoMeas2_Mito_3_01_256	log2(max(x)-x+0.01)
Nuc_Shape_Zernike_9_5	x	Cel_Int_IntegratedInt_CGAN	log2(x+1)	Cyt_Txtr_AngularScndMoment_CGAN_3_00_256	log2(x+1)	Cyt_Txtr_InfoMeas2_Mito_3_02_256	log2(max(x)-x+0.01)
Nuc_Shape_Zernike_9_7	x	Cel_Int_IntegratedInt_EndoRet	log2(x+1)	Cyt_Txtr_AngularScndMoment_CGAN_3_01_256	log2(x+1)	Cyt_Txtr_InfoMeas2_Mito_3_03_256	log2(max(x)-x+0.01)
Nuc_Shape_Zernike_9_9	x	Cel_Int_IntegratedInt_Mito	log2(x+1)	Cyt_Txtr_AngularScndMoment_CGAN_3_02_256	log2(x+1)	Nuc_Txtr_InfoMeas1_Nuc_3_00_256	log2(max(x)-x+0.5)
Cel_Neighbors_NNeighbors_Adjacent	x	Cel_Int_LowerQInt_CGAN	log2(x+1)	Cyt_Txtr_AngularScndMoment_CGAN_3_03_256	log2(x+1)	Nuc_Txtr_InfoMeas1_Nuc_3_01_256	log2(max(x)-x+0.5)
Cel_Neighbors_PctTouching_Adjacent	x	Cel_Int_LowerQInt_EndoRet	log2(x+1)	Cyt_Txtr_AngularScndMoment_EndoRet_3_00_256	log2(x+1)	Nuc_Txtr_InfoMeas1_Nuc_3_02_256	log2(max(x)-x+0.5)
Cyt_Txtr_Corr_CGAN_3_00_256	x	Cel_Int_LowerQInt_Mito	log2(x+1)	Cyt_Txtr_AngularScndMoment_EndoRet_3_01_256	log2(x+1)	Nuc_Txtr_InfoMeas1_Nuc_3_03_256	log2(max(x)-x+0.5)
Cyt_Txtr_Corr_CGAN_3_01_256	x	Cel_Int_MADInt_CGAN	log2(x+1)	Cyt_Txtr_AngularScndMoment_EndoRet_3_02_256	log2(x+1)	Nuc_Txtr_InfoMeas2_Nuc_3_00_256	log2(max(x)-x+0.01)
Cyt_Txtr_Corr_CGAN_3_02_256	x	Cel_Int_MADInt_EndoRet	log2(x+1)	Cyt_Txtr_AngularScndMoment_EndoRet_3_03_256	log2(x+1)	Nuc_Txtr_InfoMeas2_Nuc_3_01_256	log2(max(x)-x+0.01)
Cyt_Txtr_Corr_CGAN_3_03_256	x	Cel_Int_MADInt_Mito	log2(x+1)	Cyt_Txtr_AngularScndMoment_Mito_3_00_256	log2(x+1)	Nuc_Txtr_InfoMeas2_Nuc_3_02_256	log2(max(x)-x+0.01)
Cyt_Txtr_Corr_EndoRet_3_00_256	x	Cel_Int_MassDisplacement_CGAN	log2(x+1)	Cyt_Txtr_AngularScndMoment_Mito_3_01_256	log2(x+1)	Nuc_Txtr_InfoMeas2_Nuc_3_03_256	log2(max(x)-x+0.01)
Cyt_Txtr_Corr_EndoRet_3_01_256	x	Cel_Int_MassDisplacement_EndoRet	log2(x+1)	Cyt_Txtr_AngularScndMoment_Mito_3_02_256	log2(x+1)	Nuc_Txtr_SumVar_Nuc_3_00_256	log2(x+0.01)
Cyt_Txtr_Corr_EndoRet_3_02_256	x	Cel_Int_MassDisplacement_Mito	log2(x+1)	Cyt_Txtr_AngularScndMoment_Mito_3_03_256	log2(x+1)	Nuc_Txtr_SumVar_Nuc_3_01_256	log2(x+0.01)
Cyt_Txtr_Corr_EndoRet_3_03_256	x	Cel_Int_MaxIntEdge_CGAN	log2(x+1)	Nuc_Txtr_AngularScndMoment_Nuc_3_00_256	log2(x+1)	Nuc_Txtr_SumVar_Nuc_3_02_256	log2(x+0.01)
Cyt_Txtr_Corr_Mito_3_00_256	x	Cel_Int_MaxIntEdge_EndoRet	log2(x+1)	Nuc_Txtr_AngularScndMoment_Nuc_3_01_256	log2(x+1)	Nuc_Txtr_SumVar_Nuc_3_03_256	log2(x+0.01)
Cyt_Txtr_Corr_Mito_3_01_256	x	Cel_Int_MaxIntEdge_Mito	log2(x+1)	Nuc_Txtr_AngularScndMoment_Nuc_3_02_256	log2(x+1)	Cyt_Txtr_SumVar_CGAN_3_00_256	log2(x+0.01)
Cyt_Txtr_Corr_Mito_3_02_256	x	Cel_Int_MaxInt_CGAN	log2(x+1)	Nuc_Txtr_AngularScndMoment_Nuc_3_03_256	log2(x+1)	Cyt_Txtr_SumVar_CGAN_3_01_256	log2(x+0.01)
Cyt_Txtr_Corr_Mito_3_03_256	x	Cel_Int_MaxInt_EndoRet	log2(x+1)	Cyt_Txtr_DiffVar_CGAN_3_00_256	log2(x+1)	Cyt_Txtr_SumVar_CGAN_3_02_256	log2(x+0.01)
Cyt_Txtr_DiffEntro_CGAN_3_00_256	x	Cel_Int_MaxInt_Mito	log2(x+1)	Cyt_Txtr_DiffVar_CGAN_3_01_256	log2(x+1)	Cyt_Txtr_SumVar_CGAN_3_03_256	log2(x+0.01)
Cyt_Txtr_DiffEntro_CGAN_3_01_256	x	Cel_Int_MeanIntEdge_CGAN	log2(x+1)	Cyt_Txtr_DiffVar_CGAN_3_02_256	log2(x+1)	Cyt_Txtr_SumVar_EndoRet_3_00_256	log2(x+0.01)
Cyt_Txtr_DiffEntro_CGAN_3_02_256	x	Cel_Int_MeanIntEdge_EndoRet	log2(x+1)	Cyt_Txtr_DiffVar_CGAN_3_03_256	log2(x+1)	Cyt_Txtr_SumVar_EndoRet_3_01_256	log2(x+0.01)
Cyt_Txtr_DiffEntro_CGAN_3_03_256	x	Cel_Int_MeanIntEdge_Mito	log2(x+1)	Cyt_Txtr_DiffVar_EndoRet_3_00_256	log2(x+1)	Cyt_Txtr_SumVar_EndoRet_3_02_256	log2(x+0.01)
Cyt_Txtr_DiffEntro_EndoRet_3_00_256	x	Cel_Int_MeanInt_CGAN	log2(x+1)	Cyt_Txtr_DiffVar_EndoRet_3_01_256	log2(x+1)	Cyt_Txtr_SumVar_EndoRet_3_03_256	log2(x+0.01)
Cyt_Txtr_DiffEntro_EndoRet_3_01_256	x	Cel_Int_MeanInt_EndoRet	log2(x+1)	Cyt_Txtr_DiffVar_EndoRet_3_02_256	log2(x+1)	Cyt_Txtr_SumVar_Mito_3_00_256	log2(x+0.01)
Cyt_Txtr_DiffEntro_EndoRet_3_02_256	x	Cel_Int_MeanInt_Mito	log2(x+1)	Cyt_Txtr_DiffVar_EndoRet_3_03_256	log2(x+1)	Cyt_Txtr_SumVar_Mito_3_01_256	log2(x+0.01)
Cyt_Txtr_DiffEntro_EndoRet_3_03_256	x	Cel_Int_MedInt_CGAN	log2(x+1)	Cyt_Txtr_DiffVar_Mito_3_00_256	log2(x+1)	Cyt_Txtr_SumVar_Mito_3_02_256	log2(x+0.01)
Cyt_Txtr_DiffEntro_Mito_3_00_256	x	Cel_Int_MedInt_EndoRet	log2(x+1)	Cyt_Txtr_DiffVar_Mito_3_01_256	log2(x+1)	Cyt_Txtr_SumVar_Mito_3_03_256	log2(x+0.01)
Cyt_Txtr_DiffEntro_Mito_3_01_256	x	Cel_Int_MedInt_Mito	log2(x+1)	Cyt_Txtr_DiffVar_Mito_3_02_256	log2(x+1)	Cyt_Txtr_Var_CGAN_3_00_256	log2(x+0.01)
Cyt_Txtr_DiffEntro_Mito_3_02_256	x	Cel_Int_MinIntEdge_CGAN	log2(x+1)	Cyt_Txtr_DiffVar_Mito_3_03_256	log2(x+1)	Cyt_Txtr_Var_CGAN_3_01_256	log2(x+0.01)
Cyt_Txtr_DiffEntro_Mito_3_03_256	x	Cel_Int_MinIntEdge_EndoRet	log2(x+1)	Nuc_Txtr_DiffVar_Nuc_3_00_256	log2(x+1)	Cyt_Txtr_Var_CGAN_3_02_256	log2(x+0.01)
Nuc_Txtr_DiffEntro_Nuc_3_00_256	x	Cel_Int_MinIntEdge_Mito	log2(x+1)	Nuc_Txtr_DiffVar_Nuc_3_01_256	log2(x+1)	Cyt_Txtr_Var_CGAN_3_03_256	log2(x+0.01)
Nuc_Txtr_DiffEntro_Nuc_3_01_256	x	Cel_Int_MinInt_CGAN	log2(x+1)	Nuc_Txtr_DiffVar_Nuc_3_02_256	log2(x+1)	Cyt_Txtr_Var_EndoRet_3_00_256	log2(x+0.01)
Nuc_Txtr_DiffEntro_Nuc_3_02_256	x	Cel_Int_MinInt_EndoRet	log2(x+1)	Nuc_Txtr_DiffVar_Nuc_3_03_256	log2(x+1)	Cyt_Txtr_Var_EndoRet_3_01_256	log2(x+0.01)
Nuc_Txtr_DiffEntro_Nuc_3_03_256	x	Cel_Int_MinInt_Mito	log2(x+1)	Nuc_Shape_Area	log2(x+1)	Cyt_Txtr_Var_EndoRet_3_02_256	log2(x+0.01)
Cyt_Txtr_Entropy_CGAN_3_00_256	x	Cel_Int_StdIntEdge_CGAN	log2(x+1)	Nuc_Shape_BBoxArea	log2(x+1)	Cyt_Txtr_Var_EndoRet_3_03_256	log2(x+0.01)
Cyt_Txtr_Entropy_CGAN_3_01_256	x	Cel_Int_StdIntEdge_EndoRet	log2(x+1)	Nuc_Shape_Compactness	log2(x+1)	Cyt_Txtr_Var_Mito_3_00_256	log2(x+0.01)
Cyt_Txtr_Entropy_CGAN_3_02_256	x	Cel_Int_StdIntEdge_Mito	log2(x+1)	Nuc_Shape_ConvexArea	log2(x+1)	Cyt_Txtr_Var_Mito_3_01_256	log2(x+0.01)
Cyt_Txtr_Entropy_CGAN_3_03_256	x	Cel_Int_StdInt_CGAN	log2(x+1)	Nuc_Shape_EquivDiameter	log2(x+1)	Cyt_Txtr_Var_Mito_3_02_256	log2(x+0.01)
Cyt_Txtr_Entropy_EndoRet_3_00_256	x	Cel_Int_StdInt_EndoRet	log2(x+1)	Cyt_Shape_Area	log2(x+1)	Cyt_Txtr_Var_Mito_3_03_256	log2(x+0.01)
Cyt_Txtr_Entropy_EndoRet_3_01_256	x	Cel_Int_StdInt_Mito	log2(x+1)	Nuc_Shape_FormFactor	log2(x+1)	Nuc_Txtr_Var_Nuc_3_00_256	log2(x+0.01)
Cyt_Txtr_Entropy_EndoRet_3_02_256	x	Cel_Int_UpperQInt_CGAN	log2(x+1)	Nuc_Shape_MajorAxisLength	log2(x+1)	Nuc_Txtr_Var_Nuc_3_01_256	log2(x+0.01)
Cyt_Txtr_Entropy_EndoRet_3_03_256	x	Cel_Int_UpperQInt_EndoRet	log2(x+1)	Nuc_Shape_MaxFeretDiameter	log2(x+1)	Nuc_Txtr_Var_Nuc_3_02_256	log2(x+0.01)
Cyt_Txtr_Entropy_Mito_3_00_256	x	Cel_Int_UpperQInt_Mito	log2(x+1)	Nuc_Shape_MaxRadius	log2(x+1)	Nuc_Txtr_Var_Nuc_3_03_256	log2(x+0.01)
Cyt_Txtr_Entropy_Mito_3_01_256	x			Nuc_Shape_MeanRadius	log2(x+1)		
Cyt_Txtr_Entropy_Mito_3_02_256	x			Nuc_Shape_MedRadius	log2(x+1)		
Cyt_Txtr_Entropy_Mito_3_03_256	x			Nuc_Shape_Perimeter	log2(x+1)		

Supplementary File 2: Cell Painting regularization procedure. Formula of transformation applied per CP feature, ordered by feature families. $2\{\}$ means that the transformation has been applied two times.
